# Complete Neurologic Recovery of Cerebral Fat Embolism Syndrome in Sickle Cell Disease

**DOI:** 10.7759/cureus.29111

**Published:** 2022-09-13

**Authors:** Oluwayomi Oyedeji, Nwabundo Anusim, Mohammad Alkhoujah, Vrushali Dabak, Zaher K Otrock

**Affiliations:** 1 Pathology and Laboratory Medicine, Henry Ford Hospital, Detroit, USA; 2 Oncology, University of Texas, Austin, USA; 3 Neurology, Henry Ford Hospital, Detroit, USA; 4 Internal Medicine, Henry Ford Hospital, Detroit, USA

**Keywords:** magnetic resonance imaging, transfusion, seizures, fat embolism syndrome, sickle cell disease

## Abstract

Sickle cell disease is one of the most common inherited hemoglobinopathies diagnosed in the United States. Patients often present with severe anemia, pain crises, infections, and vaso-occlusive phenomena. Complications of these disorders can lead to significant debilitating morbidity and mortality. Fat embolism syndrome (FES) is a rare and devastating complication of sickle cell disease. It usually presents with a rapidly deteriorating clinical course, and the prognosis is dismal. We report a case of FES in a 19-year-old African American male with a history of sickle cell disease who presented with tonic-clonic seizures and was found to have multi-organ failure. FES was diagnosed 20 days from a presentation based on blood cytopenias and magnetic resonance imaging findings that were obscured at the initial presentation. We describe in this report, the patient’s course from presentation until diagnosis and resolution. Our case is peculiar as the patient had a very good outcome without the need for red blood cell (RBC) exchange; instead, supportive treatment and simple RBC transfusions were enough to change the clinical course of this almost fatal syndrome.

## Introduction

Fat embolism syndrome (FES) is an infrequent complication that occurs following the release of fat emboli into the systemic circulation, mostly described after long bone fractures and post-orthopedic surgeries [1]. It has been described well in trauma patients. However, FES is incompletely understood in non-trauma patients; both biochemical and mechanical mechanisms have been proposed to explain its pathophysiology [2]. It is probably the most devastating complication of sickle cell disease resulting from bone marrow necrosis. It is often under-recognized, and many cases are diagnosed at autopsy [3,4]. FES can result in the classic triad (Bergman's triad) of progressive respiratory distress, neurologic abnormalities, and skin or mucosal petechiae. Other less common findings include fever, tachycardia, renal and retinal changes, jaundice, sudden drop in hemoglobin (Hgb) and platelet count, and high erythrocyte sedimentation rate [4,5]. Diagnosis of FES is challenging as there is no benchmark test. Its diagnosis relies on the combination of clinical symptoms and laboratory and imaging findings [6]. It usually presents with a rapidly deteriorating clinical course, and the prognosis is dismal. Treatment is primarily supportive to maintain oxygenation, support hemodynamics, and resuscitating with fluids and blood components as needed [1].

The current case illustrates the neurologic manifestations and clinical course of FES in sickle cell disease. The patient presented with seizures and shortness of breath and had a complicated clinical course but had resolution of neurologic deficits with supportive management only.

## Case presentation

A 19-year-old African American male with a history of sickle cell disease presented in March 2018 to the Emergency Department after having an episode of seizure activity lasting one minute. In the week prior to the seizure, the patient has been complaining of bilateral leg pain not relieved with pain medications and hydration. There was no prior history of seizures, and his last sickle pain crisis was three months prior to presentation. In the Emergency Department, he was febrile (temperature of 101 degrees F) with a respiratory rate of 28/min and oxygen saturation of 91%. On physical examination, he was not in distress. Mental state examination showed impaired attention, concentration, and memory with impaired orientation to place, person, and time. The Glasgow coma scale was 8/15. Cranial nerve examination was normal, and motor examination was limited due to a patient not following commands but overall symmetric, with symmetric reflexes and response to painful stimuli. Laboratory workup was significant for leukocytosis (white blood cell count (WBC) 18.1 x 10^3^/µL with a neutrophil predominance of 63%), anemia (Hgb 7.3 g/dL) with elevated reticulocyte count, and hyponatremia (Na 121 mEq/L). Platelet count (211 x10^3^/µL) and creatinine (0.59 mg/dL) were within normal. Peripheral smear showed schistocytes and sickle cells with no features suggestive of leukemoid reaction. On diagnostic imaging, a chest x-ray showed no acute cardiopulmonary process. Computed tomography (CT) scan of the head without intravenous contrast showed no acute intracranial process. CT angiography of the head and neck showed no intracranial aneurysm, occlusion, or flow-limiting stenosis. The patient’s clinical status worsened and was subsequently intubated for airway protection, started on levetiracetam, and transferred to the Intensive Care Unit.

**Figure 1:**
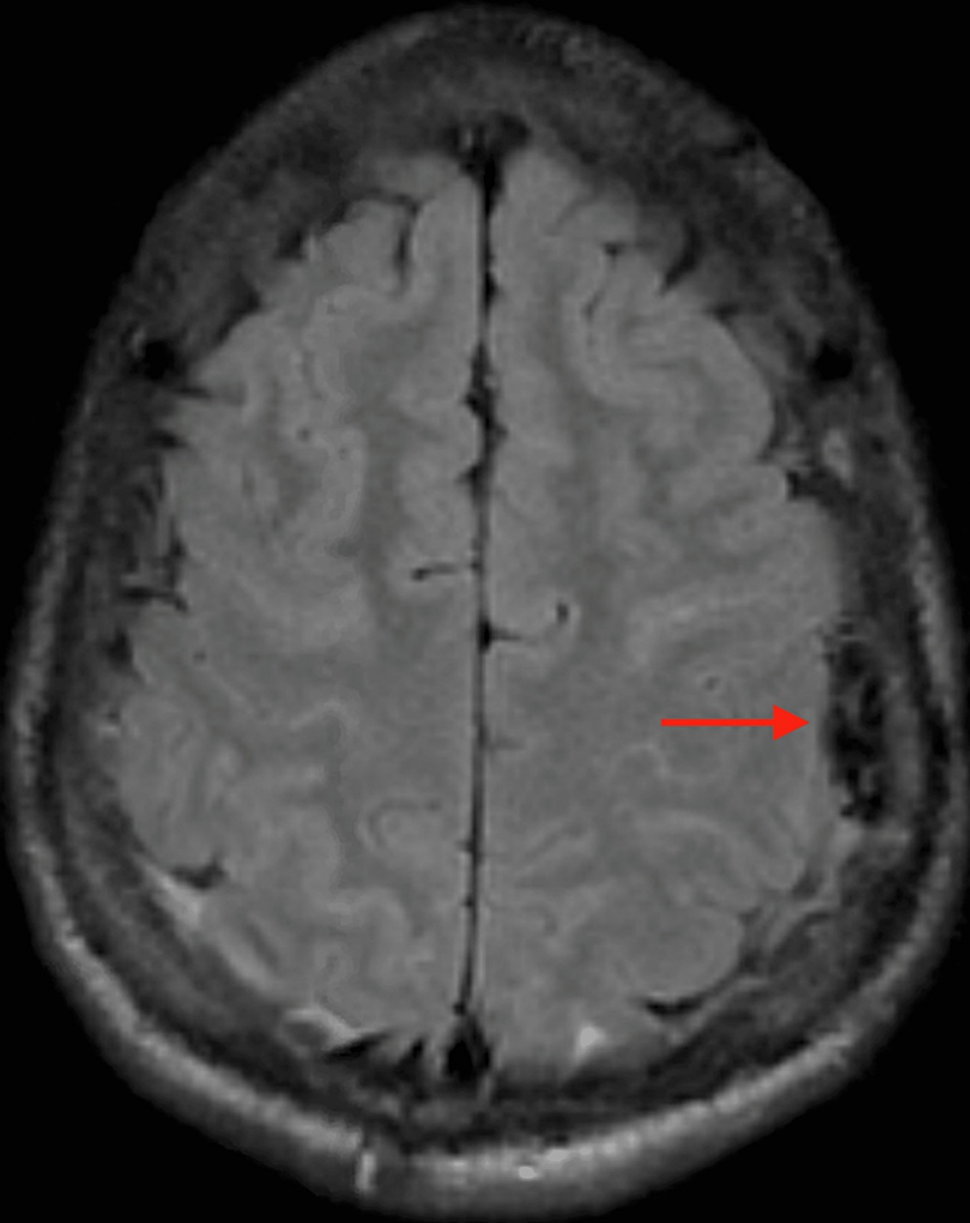
Axial T2 flair MRI showing left parietal subdural hematoma (arrow)

Lumbar puncture was performed, and the patient was started on empiric antimicrobials with ceftriaxone, vancomycin, and acyclovir. Hemoglobin evaluation revealed an HgbS of 93.3%. The patient was transfused with two units of packed red blood cells (RBCs) as he was hypotensive and tachycardic. He developed multi-organ failure manifesting with worsening renal function, elevated liver enzyme levels, elevated creatine phosphokinase, and up-trending troponins. A cardiac echocardiogram showed evidence of right-sided heart strain with high suspicion of pulmonary embolism and hence he was started on high-intensity heparin. While on heparin patient repeatedly had tonic-clonic seizures which necessitated magnetic resonance imaging (MRI) evaluation without gadolinium showing subdural hematoma (Figure 1).

Heparin was immediately discontinued. Routine electroencephalogram revealed near continuous 2 Hz frontally predominant generalized rhythmic delta activity with triphasic morphology indicative of moderate encephalopathy, with no epileptiform discharges. The patient required multiple RBC transfusions to correct his worsening anemia. The patient’s WBC count normalized on day 3. A ventilation-perfusion scan showed a low probability of pulmonary embolism. Red cell exchange was discussed among the managing teams and was agreed to be not indicated given the lack of evidence of acute chest syndrome or ischemic stroke. In addition, a repeat Hgb evaluation revealed a drop in HgbS to 25% due to repeated blood transfusions. On day 5 of admission, the patient had worsening blood work showing pancytopenia; his WBC count dropped from 5.1 x 10^3^ to 2.6 x 10^3^/µL, hemoglobin dropped from 8.3 to 5 g/dL, and platelet count decreased from 129 x 10^3^ to 38 x 10^3^/µL. However, the patient remained hemodynamically stable. CT scan of the chest and abdomen did not show evidence of bleeding. Hemolysis workup showed an increase in lactate dehydrogenase. A peripheral blood smear revealed microcytic hypochromic anemia with marked anisopoikilocytosis including sickle cells and schistocytes. Blood and cerebrospinal fluid cultures took on admission did not grow bacterial or fungal organisms. Supportive management with inotropes and transfusions was maintained.

The patient’s mental status improved gradually, and he was extubated on day 9. His clinical course was complicated by pneumothorax requiring placement of a chest tube, methicillin-sensitive Staphylococcus aureus bacteremia treated with cefazolin, and critical illness myopathy. The patient remained hemodynamically stable and did not require mechanical ventilation. He was still not oriented but intermittently following commands, with his physical examination showing symmetric proximal muscle weakness (the patient was unable to move antigravity and he had 2/5 strength in the proximal muscles in the upper and lower extremities).

**Figure 2:**
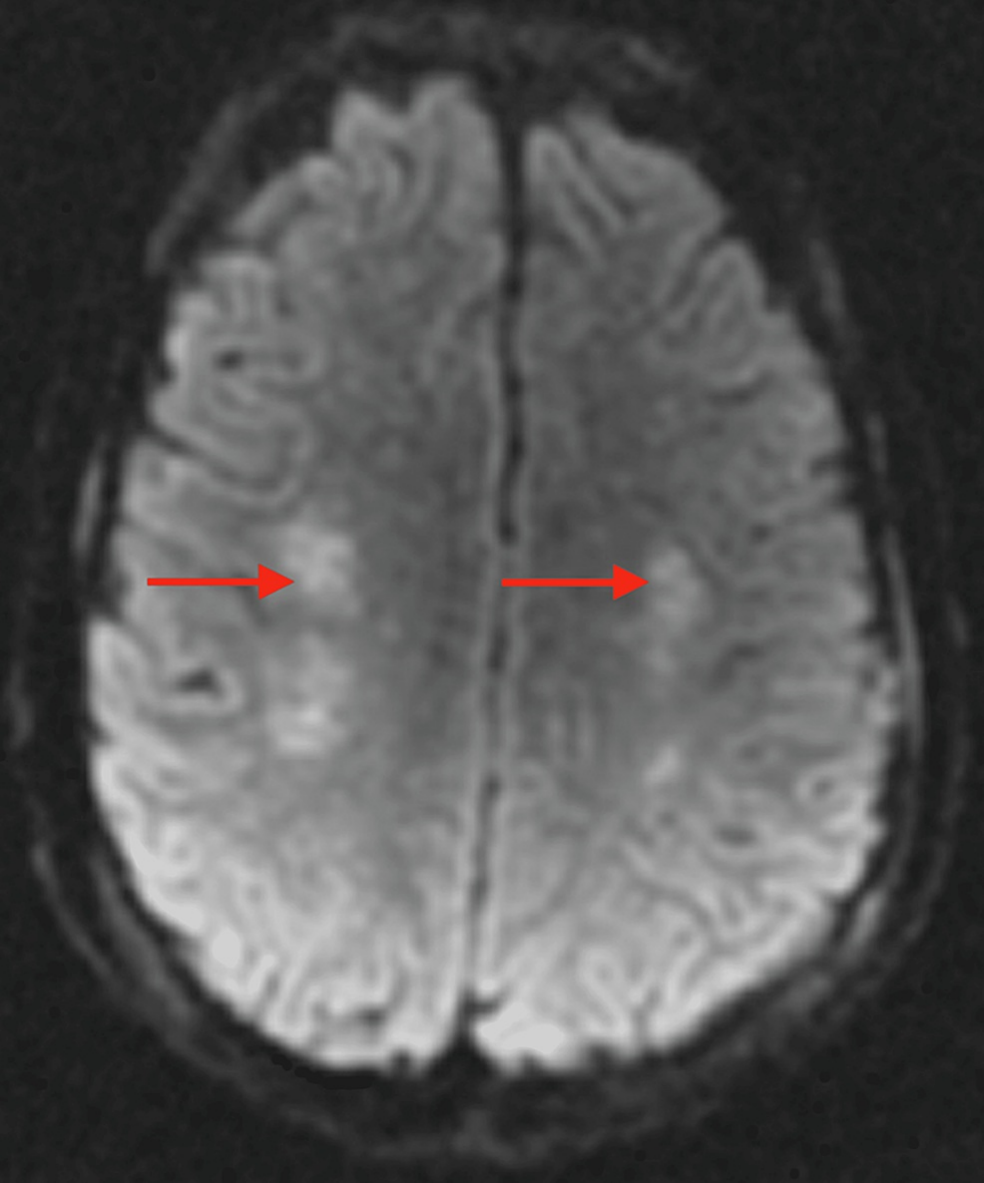
Axial DWI sequence of follow-up MRI showing symmetric bilateral diffusion restriction within the centrum semiovale (arrows)

**Figure 3:**
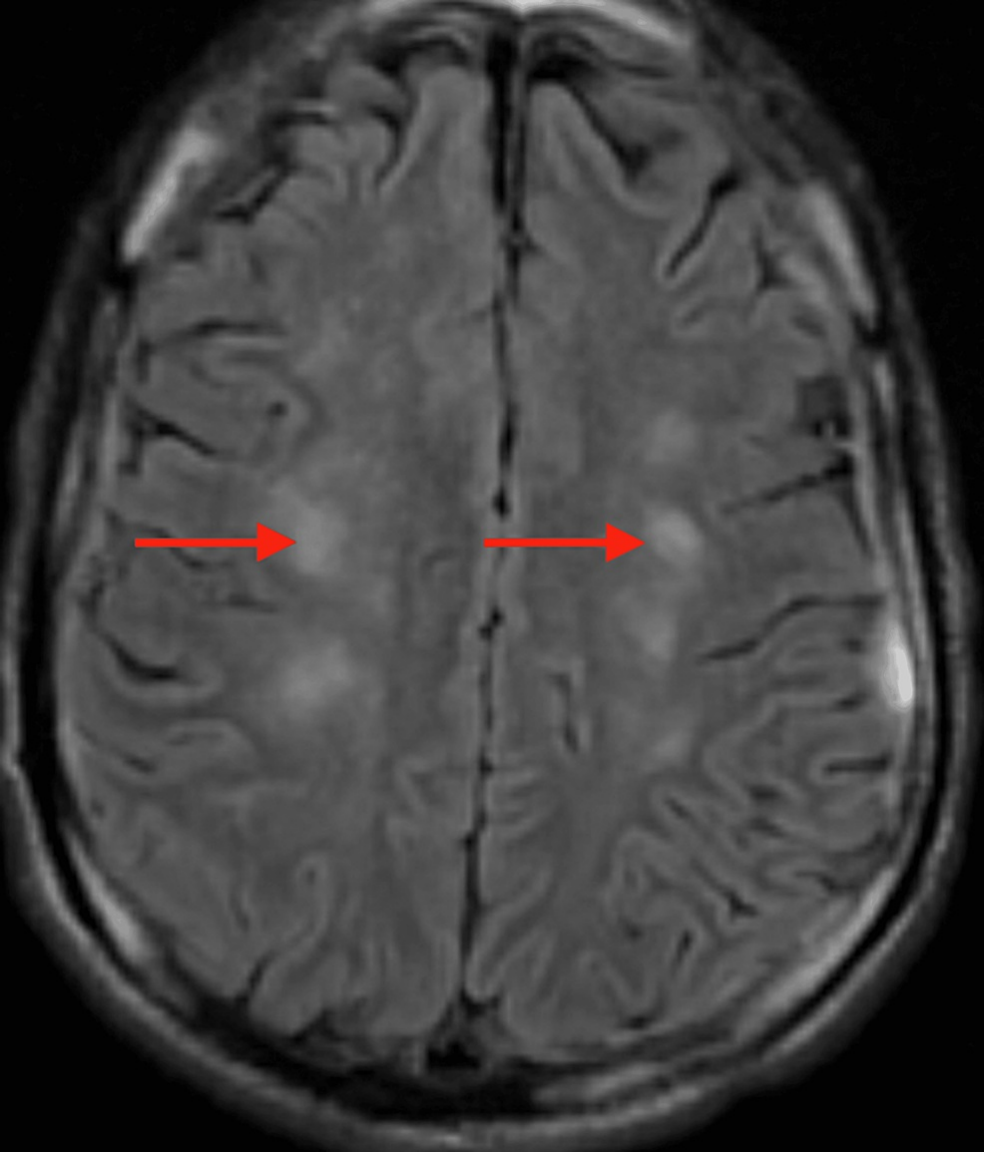
Axial T2 flair changes (arrows) correlating with diffusion restriction

**Figure 4:**
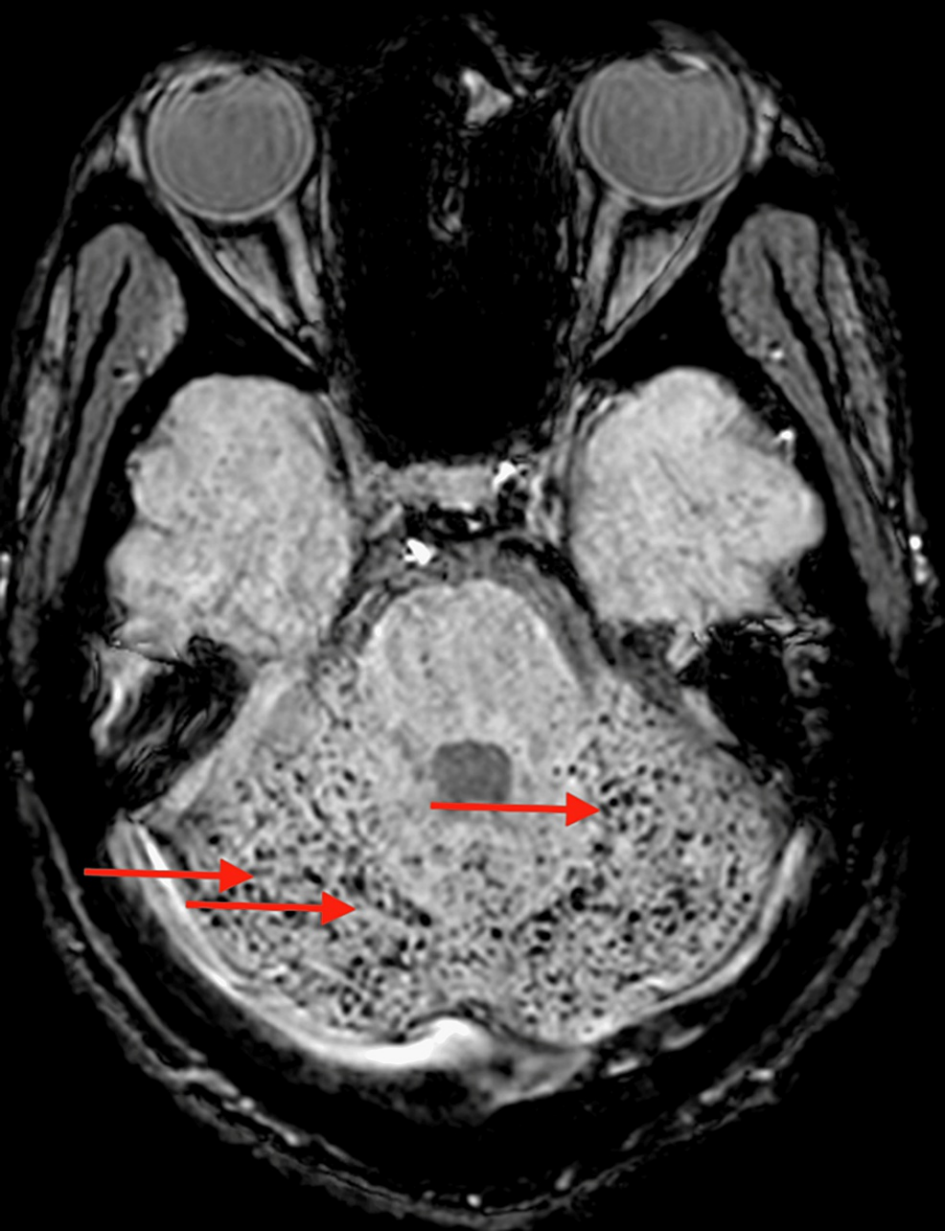
Axial MRI SWI sequence showing innumerable foci of signal attenuation in the posterior fossa consistent with “starfield” appearance of fat embolism

**Figure 5:**
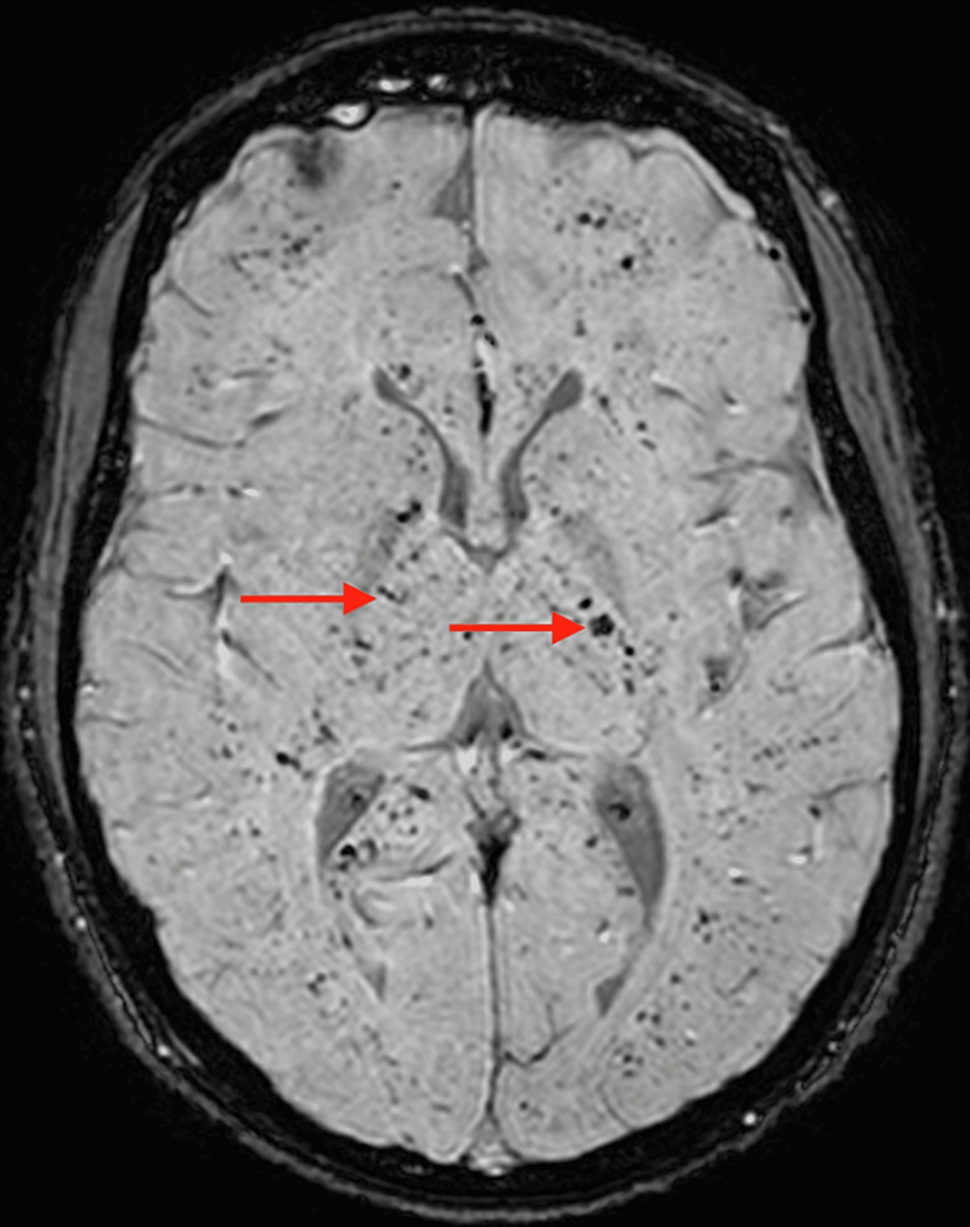
Axial MRI SWI sequence showing innumerable foci of signal attenuation in the descending cortical tracts consistent with “starfield” appearance of fat embolism

**Figure 6:**
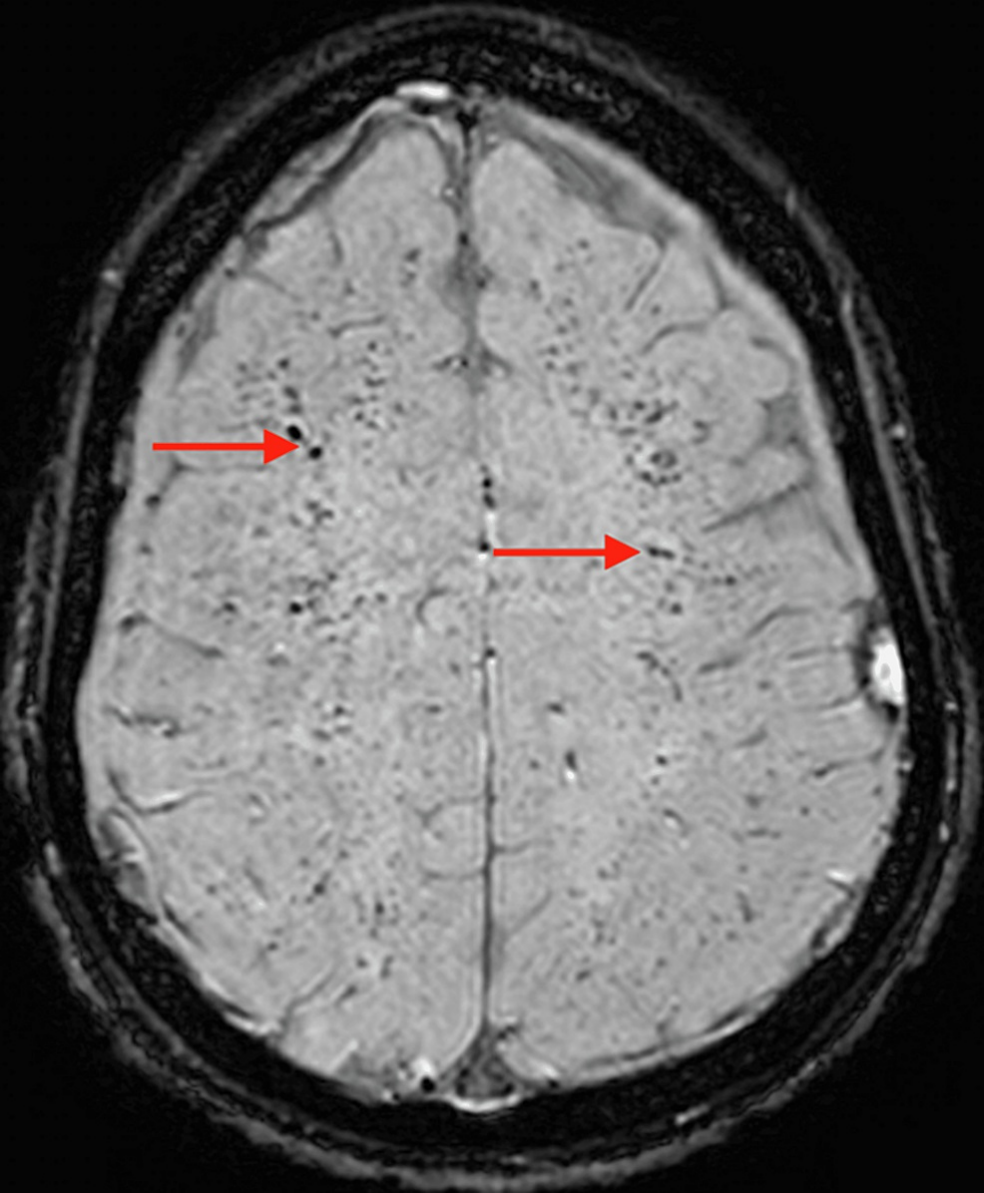
Axial MRI SWI sequence showing innumerable foci of signal attenuation in the subcortical white matter consistent with “starfield” appearance of fat embolism

The patient continued to improve gradually, and he was transferred to the general medicine ward on day 11. His nasogastric tube was removed, weaned off anti-seizure medications, and he was able to participate actively in physical therapy. A repeat brain MRI without contrast showed bilateral symmetric diffusion restriction (Figure 2) and T2/flair hyperintensities in the supratentorium subcortically (Figure 3) with concern for possible fat emboli syndrome. A follow-up MRI with contrast was highly suggestive of fat emboli syndrome; susceptibility weighted imaging confirmed innumerable punctate foci of signal attenuation in the posterior fossa (Figure 4), descending cortical spinal tracts (Figure 5), and subcortical white matter (Figure 6).

 

 

 

 

The patient continued to spike fever with a negative workup. Repeat lumbar puncture showed no infectious process. Upper extremity Doppler ultrasound scan showed right deep venous thrombosis; however, initiation of anticoagulation was deferred given the risk of bleeding.

He was discharged to a rehabilitation facility on day 25 in stable condition. The patient was followed up in the neurology clinic after three months with no further seizure activity; his physical examination showed intact higher cortical functioning with significant improvement in his muscle weakness (4+/5 in proximal muscles in upper and lower extremities). He was followed up with his primary care physician one and three years post his admission with the patient going back to his baseline mentation, function, and strength.

## Discussion

We described the case of a 19-year-old male with Hgb SS disease presenting with seizures, decreased level of consciousness, and shortness of breath following a pain crisis. His clinical course was complicated by multi-organ failure which could have been triggered by his seizures causing rhabdomyolysis and elevated creatine phosphokinase. He was later diagnosed with cerebral FES based on clinical and radiologic findings. The patient was able to recover from this nearly fatal complication with supportive measures only.

Sickle cell disease is one of the most common inherited disorders in the United States affecting one in 600 African Americans [7]. The disease is characterized by the presence of hemoglobin S which typically results from a point mutation in the beta globin gene that leads to the substitution of a valine for glutamic acid in position 6. The disease can be inherited as a homozygous disorder (Hgb SS) or as a heterozygous disorder in combination with another mutation occurring in the beta-globin gene such as hemoglobin S thalassemia, hemoglobin SD, hemoglobin SE, and hemoglobin SC [8]. Of the sickle cell diseases, hemoglobin SS and SC variants are the most common types. Patients with hemoglobin SS have comparable clinical manifestations with hemoglobin S beta0 thalassemia and more severe manifestations of vaso-occlusive crises than those with hemoglobin SC disease [9,10].

Sickle cell patients in pain crises typically present due to occlusion of capillaries and small blood vessels by the sickle cells. Symptoms can be triggered by an infection or dehydration; however, it is also not uncommon to have no identifiable precipitating events. Common presentations of vaso-occlusive disease include ischemic stroke, bone pain, and acute chest syndrome. Other forms of debilitating crises are aplastic anemia, acute sequestration, and hyper-hemolytic crises [11]. Without the institution of appropriate early management care, most sickle cell anemia patients will have chronic organ damage and overall reduced life expectancy [12]. FES is rarely seen in sickle cell patients [3,4]. The diagnosis is challenging and can be delayed due to its rarity and its similarities with other clinical conditions such as multi-organ failure or thrombotic thrombocytopenic purpura [4]. The pathogenesis of this syndrome is unknown, and several theories exist. It is thought to be caused by bone marrow necrosis resulting in the release of fat globules into the venous system and subsequently the arteriolar system through a patent foramen ovale or other anatomical shunts into the arterial circulation [3]. Although structural obstruction by the fat emboli is necessary for the initiation and occurrence of FES, this may not be a sufficient requirement because it has also been hypothesized that biochemical elements, such as agglutination of chylomicrons and very-low-density lipoprotein cholesterol and direct damage to tissues by free fatty acids, might have essential roles in the pathogenesis of this syndrome [13,14].

FES in clinical settings can easily go under-recognized within the spectrum of manifestation of vaso-occlusive episodes. Its diagnosis is primarily clinically supported by laboratory and imaging findings [6]. The mortality rate is high, up to 66%. The common clinical presentation is that of a painful vaso-occlusive crisis with unusual severity [15]. The triad of respiratory distress, neurologic symptoms, and skin petechiae has been described in the literature. In addition, patients have evidence of multi-organ failure [16]. When CNS involvement is suspected in FES, an MRI of the brain should be obtained which may reveal a “starfield” pattern [17]. However, according to Kuo et al., several different imaging patterns exist in patients with cerebral fat embolism and are dependent on the timing of the imaging [18].

Supportive care is usually the mainstay of treatment for FES. Reduction of hemoglobin S by simple transfusions and manual or automated RBC exchange has been reported to be effective in alleviating symptoms [1,15,19]. Tsitsikas and colleagues reported a better survival rate with exchange transfusion in a retrospective study of eight patients, and they concluded that RBC exchange “should be instituted as soon as FES is suspected” [15]. It is worth mentioning that FES in sickle cell disease has not yet been listed as an indication for RBC exchange according to the most recent American Society for Apheresis (ASFA) 2019 guidelines [20]. The patient was managed with a simple blood transfusion on presentation to correct for anemia and improve the oxygen-carrying capacity [21]. Our team considered RBC exchange as a treatment option after the patient developed multi-organ failure. However, the decision was to hold on to RBC exchange for three main reasons: 1) The patient was not hemodynamically stable and RBC exchange was associated with many potential risks; 2) RBC exchange in the setting of sickle cell disease with multi-organ failure is a category III indication according to ASFA guidelines, which means the “optimum role of apheresis therapy is not established” and the “decision making should be individualized”; and 3) The repeat hemoglobin evaluation revealed a drop in HgbS to 25% due to repeated blood transfusions which made RBC exchange of no additional benefit.

Our patient was homozygous for hemoglobin S with 93.3% at presentation. He presented with respiratory and neurologic symptoms and a rapidly progressive course with multi-organ failure. During his admission, he manifested worsening thrombocytopenia, anemia, hemolysis, and schistocytes on peripheral smear which can be part of bone marrow aplasia complicating sickle cell disease and can also be confused with thrombotic thrombocytopenic purpura. The initial MRI was not specific, and a repeat MRI 14 days later was highly suggestive of FES. Our patient was transfused with several units of packed RBCs with a reduction in HgbS from 93.3% to 25%. He recovered without the need for an RBC exchange with a resolution of neurologic symptoms.

## Conclusions

There should be a high level of suspicion for FES in patients with sickle cell disease. Signs that might alert a physician to this diagnosis include severe painful vaso-occlusive crisis, neurologic symptoms, and radiologic findings on MRI. Our case is peculiar as the patient had a very good outcome without the need for RBC exchange; instead, supportive treatment and RBC transfusion were enough to change the clinical course of this almost fatal syndrome. It is important to mention that the treatment decisions, in this case, were based on the patient’s clinical course and the priority choices made collaboratively among the medical teams participating in his care.
